# A Machine Learning Framework for Cognitive Impairment Screening from Speech with Multimodal Large Models

**DOI:** 10.3390/bioengineering13010073

**Published:** 2026-01-08

**Authors:** Shiyu Chen, Ying Tan, Wenyu Hu, Yingxi Chen, Lihua Chen, Yurou He, Weihua Yu, Yang Lü

**Affiliations:** 1 Laboratory of Research and Translation for Geriatric Diseases, Department of Geriatrics, The First Affiliated Hospital of Chongqing Medical University, Youyi Road, Yuzhong District, Chongqing 400016, China; 2023140102@stu.cqmu.edu.cn (S.C.); taryn_pjt@163.com (Y.T.); 2020110153@stu.cqmu.edu.cn (L.C.); 2023110047@stu.cqmu.edu.cn (Y.H.); 2 Tianfu Jiangxi Laboratory, Chengdu 641400, China; hwyuestc@gmail.com; 3 Institute of Neuroscience, Chongqing Medical University, Chongqing 400016, China; chenyingxicqmu@163.com

**Keywords:** Alzheimer’s disease, digital biomarkers, acoustic feature extraction, machine learning, early diagnosis

## Abstract

**Background**: Early diagnosis of Alzheimer’s disease (AD) is essential for slowing disease progression and mitigating cognitive decline. However, conventional diagnostic methods are often invasive, time-consuming, and costly, limiting their utility in large-scale screening. There is an urgent need for scalable, non-invasive, and accessible screening tools. **Methods:** We propose a novel screening framework combining a pre-trained multimodal large language model with structured MMSE speech tasks. An artificial intelligence-assisted multilingual Mini-Mental State Examination system (AAM-MMSE) was utilized to collect voice data from 1098 participants in Sichuan and Chongqing. CosyVoice2 was used to extract speaker embeddings, speech labels, and acoustic features, which were converted into statistical representations. Fourteen machine learning models were developed for subject classification into three diagnostic categories: Healthy Control (HC), Mild Cognitive Impairment (MCI), and Alzheimer’s Disease (AD). SHAP analysis was employed to assess the importance of the extracted speech features. **Results:** Among the evaluated models, LightGBM and Gradient Boosting classifiers exhibited the highest performance, achieving an average AUC of 0.9501 across classification tasks. SHAP-based analysis revealed that spectral complexity, energy dynamics, and temporal features were the most influential in distinguishing cognitive states, aligning with known speech impairments in early-stage AD. **Conclusions:** This framework offers a non-invasive, interpretable, and scalable solution for cognitive screening. It is suitable for both clinical and telemedicine applications, demonstrating the potential of speech-based AI models in early AD detection.

## 1. Introduction

As the global population ages, dementia has become a pressing public health issue, significantly affecting the physical and mental well-being of elderly [1]. Among dementia types, Alzheimer’s disease (AD) is the most common, accounting for 60% to 80% of all cases [2]. AD is a progressive neurodegenerative disorder primarily affecting individuals over 65 years old, marked by symptoms such as memory loss, language impairment, executive dysfunction, spatial disorientation, and changes in personality and behavior [3]. Language dysfunction, a hallmark of AD, involves deficits in both motor control of speech production and higher-level cognitive functions like language planning, semantic organization, and logical reasoning [3,4].

The onset of AD is typically preceded by an intermediate stage known as mild cognitive impairment (MCI), particularly the amnestic subtype (aMCI), which is regarded as a potential prodromal phase of AD [5]. Early identification of MCI is clinically significant for delaying cognitive decline, developing intervention strategies, and prolonging quality-adjusted life years [6]. However, diagnosing AD and MCI remains challenging due to their subtle onset and slow progression. While neuroimaging and biomarker such as PET scans and cerebrospinal fluid assays provide insight into AD pathology, their high cost, complexity, and limited accessibility restrict their routine use [7,8]. In contrast, neuropsychological scales such as the Mini-Mental State Examination (MMSE) offer advantages of simplicity and low cost, making them the mainstream tools for cognitive assessment [9,10]. However, these methods’ diagnostic accuracy is affected by factors like evaluator expertise, resource demands, and demographics such as education. Additionally, a ceiling effect limits their use across diverse populations [11,12,13].

Recently, speech has emerged as a natural, non-invasive, cost-effective, and easily collectible digital biomarker showing remarkable potential for early detection of cognitive impairment [14]. Studies have documented multiple speech abnormalities in AD patients, such as imprecise articulation, slowed speech rate, irregular rhythm, monotonous pitch, and increased pausing [15,16,17]. These changes arise not only from motor system degeneration but also from diminished cognitive resources leading to impaired language organization [18]. Such anomalies can be quantified via audio signal processing techniques across multiple dimensions, including speech rate, pause ratio, spectral energy distribution, voice perturbation parameters, spectral contrast, and entropy, laying the foundation for constructing acoustically sensitive cognitive biomarkers [19,20]. Consequently, speech analysis has become an increasingly important research direction for intelligent cognitive screening.

Although early studies suggest speech signals can help detect cognitive impairments, current methods still struggle with feature modeling, algorithm robustness, and clinical interpretability. CosyVoice2 embeddings offer high-dimensional representations of speech that encode a broad range of acoustic and temporal information potentially related to cognitive status. Conventional low-dimensional handcrafted features, including MFCCs, pitch, and formant-based measures, have been widely used in prior speech-based cognitive studies; however, their performance may vary across datasets and clinical settings. In the present study, we therefore focus on exploring the utility of large-scale learned speech representations within a unified classification framework, without aiming to provide a direct empirical comparison with traditional acoustic features [21,22]. While these features capture certain vocal characteristics, they are limited in their ability to represent the complex cognitive processes underlying language organization and semantic production [23,24]. Most existing studies that use speech to assess cognitive impairment are based on limited sample sizes or rely primarily on cohorts from Western populations [25]. These models typically perform well only in monolingual or standard-accent environments and lack generalizability to dialectal or multilingual settings [26]. Furthermore, the lack of standardized speech tasks leads to heterogeneity across studies, weakening the link between speech features and clinical assessments such as MMSE and MoCA, and reducing model interpretability and clinical utility [27].

To address these challenges, this study proposes a novel speech-based screening framework for Alzheimer’s disease that integrates high-dimensional embedded speech feature extraction using a pre-trained large language model with multiple machine learning algorithms for classification. A standardized Mini-Mental State Examination speech corpus was collected from participants in Sichuan and Chongqing, comprising three groups: healthy controls (HC), individuals with MCI, and patients with AD. For the first time in this context, the speech feature extraction module of the pre-trained large language model CosyVoice2 was first utilized. The high-dimensional speaker embeddings, speech tokens, and acoustic representations extracted by CosyVoice2 were subsequently transformed into interpretable statistical acoustic parameters, including Num frames, Mean energy, Std energy, Max energy, Energy range, Skew energy etc. These features preserve traditional acoustic characteristics while enhancing the representation of temporal structure and cognitive load, resulting in an acoustic feature space with improved cross-linguistic robustness and cognitive sensitivity.

In classification modeling, fourteen widely used classifiers such as SVM, KNN, Random Forest, XGBoost, LightGBM, Gradient Boosting, and logistic regression were systematically evaluated on a three-class classification task (HC versus MCI versus AD), employing five-fold cross-validation to ensure model robustness. The experimental results demonstrate that multiple algorithms achieve excellent performance in key metrics such as AUC and F1-score, with LightGBM and Gradient Boosting models reaching an average three-class AUC of 0.9501, significantly outperforming baseline models based on traditional low-dimensional acoustic features.

This study addresses limitations in current speech-based cognitive screening methods, including low feature interpretability, poor model generalizability, and suboptimal evaluation strategies. It proposes a novel modeling framework leveraging acoustic embeddings derived from large language models. Specifically, the CosyVoice2 feature extraction module is employed to capture high-dimensional acoustic-cognitive features from Sichuan dialect speech. These features are integrated with multiple machine learning models to classify healthy controls (HC), mild cognitive impairment (MCI), and Alzheimer’s disease (AD), leading to enhanced interpretability and cross-task adaptability. Overall, this work advances the current speech-based cognitive screening paradigm and provides a methodological foundation for the interdisciplinary integration of language neuroscience and artificial intelligence.

## 2. Methods

### 2.1. Study Participants

From 1 January 2023 to 31 December 2024, a total of 1098 patients from the Memory Clinic of the Geriatrics Department at the First Affiliated Hospital of Chongqing Medical University were screened and enrolled in this study. All participants underwent comprehensive medical history assessments, routine blood tests, neuropsychological evaluations, and structural magnetic resonance imaging (sMRI). The diagnosis of Alzheimer’s disease (AD) was based on the 2011 revised criteria for probable AD dementia established by the National Institute on Aging and the Alzheimer’s Association (NIA/AA). The diagnosis of mild cognitive impairment (MCI) followed the corresponding NIA/AA diagnostic guidelines [28]. None of the participants had a history of mental illness or prior use of cognitive-enhancing medications.

### 2.2. Ethical Considerations

This study and its informed consent were approved by medical ethics Chongqing First Affiliated Hospital Committee Medical University (Approval number: 20212901; Time of Ethical Approval: 10 May 2021). All participants or theirs. The legal guardian signs the informed consent upon receipt a detailed description of this study.

### 2.3. Automated Multilingual Cognitive Assessment and Speech-Based Classification Pipeline

**Figure 1:**
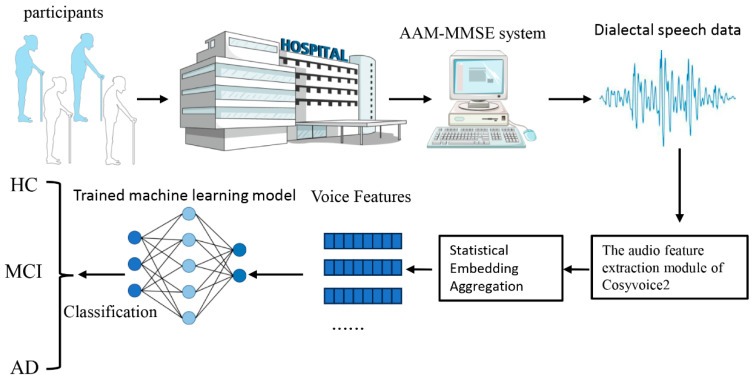
Automated Multilingual Cognitive Assessment and Speech-Based Classification Pipeline.

To reduce the clinical manpower demands and enhance the scalability of cognitive screening, this study implemented an artificial intelligence-assisted multilingual Mini-Mental State Examination system (AAM-MMSE). As shown in Figure 1, the system enabled patients to independently complete standardized cognitive assessment tasks through human–computer voice interaction, without requiring real-time supervision by medical professionals. During the assessment, patients receive voice prompts for MMSE questions via a computer interface and respond verbally. The system’s built-in automatic speech recognition (ASR) module records and transcribes responses in real time, facilitating a full automated assessment process. This approach ensure consistent administration and minimized reliance on clinical resources. Following the AAM-MMSE interaction, all recorded speech data were processed using the CosyVoice2 acoustic feature extraction module [29]. High-dimensional, frame-level acoustic embedding features were extracted from each patient’s audio responses. To generate a fixed-length, compact representation for each individual, we applied the Statistical Embedding Aggregation method, calculating statistical descriptors such as the mean and standard deviation across all frame-level features. These feature vectors were subsequently input into machine learning models to classify participants into one of three cognitive categories: cognitively normal (HC), mild cognitive impairment (MCI), or Alzheimer’s disease (AD).

### 2.4. Speech Data Collection

Previous speech data collection protocols often used the “cookie theft” picture description tasks, which required participants to generate free-form speech based on image content [30,31]. However, this method imposed significant demands on language organization and comprehension, making it difficult for individuals with moderate to severe cognitive impairments to participate effectively. Consequently, the quality of the speech data varied widely. Moreover, traditional methods typically required healthcare staff to manually operate recording devices, increasing operational workload and introducing potential artifacts such as incomplete recordings and background noise.

In this study, we utilized our self-developed AAM-MMSE system during participants’ initial clinical visits [32]. The system enabled automatic and real-time collection of complete verbal responses during the formal administration of the MMSE. As a widely used clinical tool for cognitive assessment, the MMSE includes a set of standardized language tasks such as orientation, immediate recall, attention, language comprehension, repetition, and naming. These tasks allow for efficient acquisition of clinically meaningful speech data without imposing additional cognitive or procedural burden on participants. All MMSE speech recordings were collected under the supervision of trained clinical personnel, and no audio files were missing at the acquisition stage. Quality control was therefore performed at the signal level rather than the file level. Recordings or segments exhibiting excessive noise or severe signal degradation that prevented reliable feature extraction were excluded during preprocessing. Mild background noise was attenuated using Deep Complex CRN (available at: https://github.com/huyanxin/DeepComplexCRN (accessed on 12 June 2025)), recordings were resampled to 16 kHz, and a 32-ms Hann window with 50% overlap was applied during short-time Fourier transform. The dataset includes 10–12 recordings per participant from multiple MMSE subtests, providing a reproducible basis for subsequent acoustic analyses. It should be noted that DeepComplexCRN was used exclusively as an explicit offline denoising step to suppress environmental background noise prior to feature extraction. In contrast, CosyVoice2 was not employed as a denoising algorithm per se, but as a representation model whose learned embeddings are inherently robust to residual noise and speaker interference.

### 2.5. Feature Extraction and Speaker Identification Using the CosyVoice2 Audio Module

**Figure 2:**
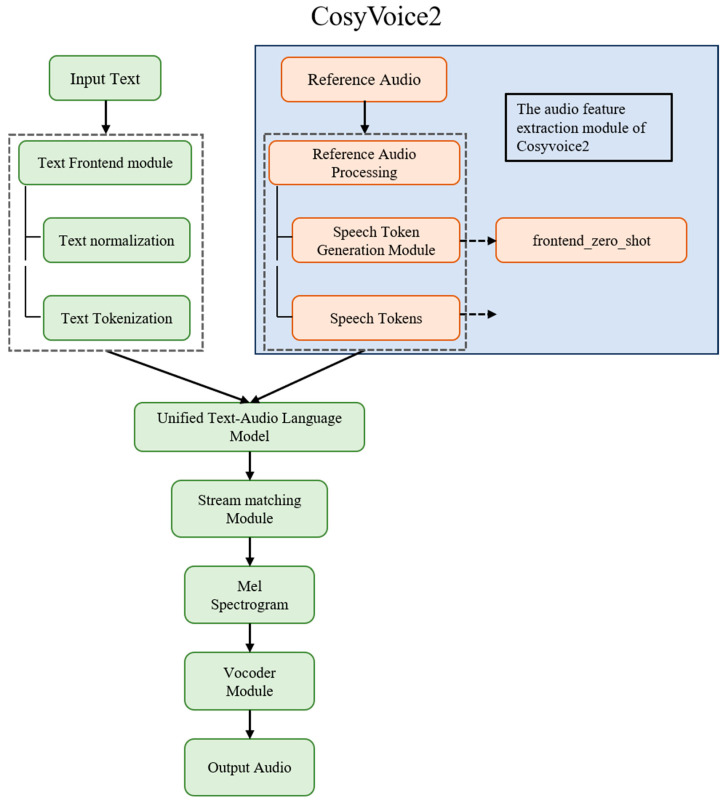
Schematic of the CosyVoice2 model architecture, with the speech feature extraction module isolated and applied in the present study.

Feature extraction and speaker identification were conducted using the audio feature extraction module of CosyVoice2 (Figure 2), a large-scale pre-trained speech model developed by Alibaba Tongyi Laboratory [29]. CosyVoice2 is primarily designed for high-fidelity, low-latency, multilingual text-to-speech (TTS) applications and is currently considered one of the most advanced speech generation models available [33]. Trained on a large-scale multilingual speech corpus, this module supports speaker identification and effectively isolates the target speaker by suppressing background noise and interfering voices. Notably, CosyVoice2 exhibits strong performance in understanding and processing regional dialects, particularly Sichuanese, with a semantic comprehension capability surpassing that of other large language models.

In this study, preprocessed MMSE audio recordings were input into the CosyVoice2 speech analysis module, which automatically extracted a set of high-dimensional speaker embeddings. Importantly, the acoustic descriptors used for classification were not conventional handcrafted features extracted directly from raw waveforms. Instead, they were statistical summaries computed from high-dimensional CosyVoice2 embeddings, and therefore represent embedding-derived acoustic features rather than traditional low-dimensional handcrafted descriptors such as MFCCs or formants. These embeddings encoded personalized speech characteristics and cognitive language features of each participant. In addition, conventional acoustic features were extracted, including Num frames, Mean energy, Std energy, Max energy, Energy range, Skew energy, etc.

Given the variable duration of individual recordings, the resulting speech features were of unequal lengths, posing challenges for model fitting and potentially compromising classification performance. To address this, statistical summarization was applied to all extracted features, transforming them into fixed-length, interpretable acoustic descriptors. These standardized features were then labeled according to the participants’ cognitive states, including HC, MCI and AD.

The speech features were extracted across several key dimensions, including mean energy (representing the average energy of the speech signal, indicative of overall vocal intensity), power sum (reflecting the total energy over time, corresponding to cumulative vocal output during the recording), and speaking ratio (the proportion of time the participant was actively speaking, used to quantify speech activity relative to pauses or silence). A comprehensive summary of all extracted features is presented in Supplementary Material Table S1.

This figure illustrates the workflow of acoustic feature extraction utilizing the pre-trained CosyVoice2 large language model. Raw audio recordings are initially fed into the CosyVoice2 speech analysis module, which performs automatic speaker identification and dialect recognition. Subsequently, high-dimensional speaker embeddings and acoustic features—Num frames, Mean energy, Std energy, Max energy, Energy range, Skew energy, etc.—are extracted. These features are then standardized and used as input for downstream machine learning tasks.

### 2.6. Establishment and Validation of Machine Learning Models

Participants were randomly assigned to a training set and an independent test set in a 6:4 ratio. Demographic characteristics are summarized in Supplementary Material Table S2. The training set was used for feature selection, model construction, and hyperparameter optimization, while the test set was reserved solely for independent performance evaluation. Class imbalance is a common challenge in clinical datasets and may adversely affect model performance, particularly for minority diagnostic groups. To mitigate this issue, the Synthetic Minority Over sampling Technique was applied to the training data using the imbalanced learn library in Python to improve class balance during model development [34]. To ensure a strict separation between training and evaluation procedures, oversampling was confined to the training data only. The dataset was initially divided into training and test sets without oversampling. Model development was conducted exclusively within the training set using stratified fivefold cross validation. In each fold, SMOTE was applied only to the training subset, whereas the validation subset remained unchanged. Following model selection and hyperparameter tuning, each classifier was retrained using the full training set without oversampling. Final model performance was evaluated on the independent test set to obtain an unbiased estimate of generalization performance.

In this study, Bayesian optimization was used to search the hyperparameters of 14 commonly employed supervised learning algorithms for classifying participants into HC, MCI, and AD groups [35]. The search ranges for key hyperparameters were defined based on prior literature and practical considerations. It should be emphasized that the aim of this study was not to establish generalizable rules or systematic patterns for optimal hyperparameter configurations, but to identify parameter settings that are relatively stable and well suited to the present dataset. A more in-depth investigation of the underlying behavior and regularities of hyperparameters is therefore beyond the scope of this work and will be pursued in future studies. All optimization procedures used balanced accuracy as the objective function and were strictly confined to the training subset within each cross-validation fold, thereby ensuring that validation data were not accessed during the tuning process. The evaluated classifiers included Logistic Regression (LR) [36], Random Forest (RF) [37], Extra Trees [38], Gradient Boosting [39], AdaBoost [40], k-Nearest Neighbors (k-NN) [41], Decision Tree [42], Support Vector Machine (SVM) [43], Naïve Bayes [44], Linear Discriminant Analysis (LDA) [45], Quadratic Discriminant Analysis (QDA) [46], eXtreme Gradient Boosting (XGBoost) [47], Light Gradient Boosting Machine (LightGBM) [48], and the Dummy classifier [49] as a baseline reference.

**Figure 3:**
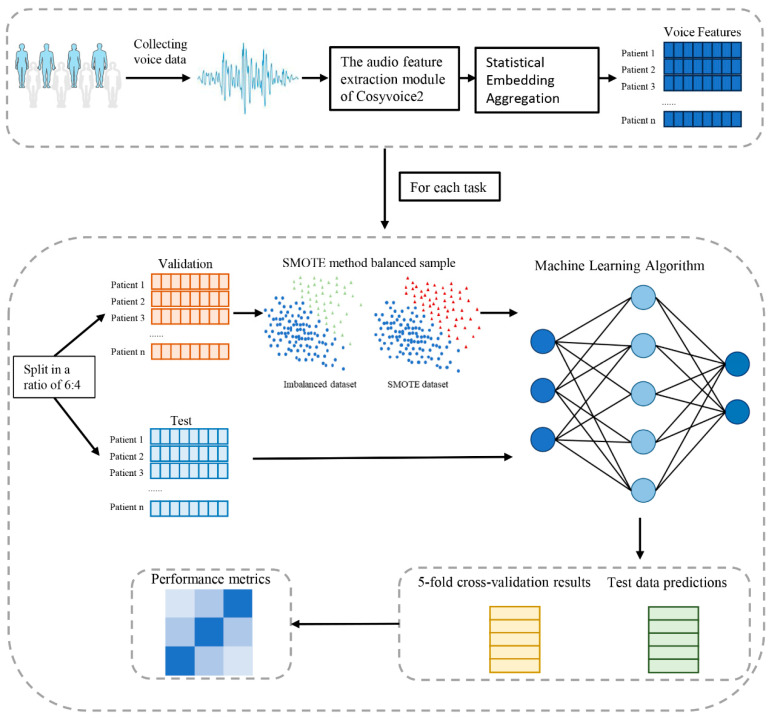
Flowchart of the proposed framework, comprising two main phases: data acquisition and pre-processing, and construction and evaluation of the classification model.

All models were implemented using the scikit-learn (sklearn) library in Python. Model performance was comprehensively assessed using standard classification metrics, including precision, recall, F1-score, and the area under the receiver operating characteristic curve (AUC). A schematic overview of the entire modeling pipeline is presented in Figure 3.

### 2.7. Feature Importance Assessment

To improve model interpretability and clarify how each feature contributes to the classification results, we applied the SHapley Additive Explanations (SHAP) method based on game theory [50]. SHAP assigns a positive or negative contribution value to each feature for every prediction, explaining individual outcomes. This value reflects how the expected model output changes when the feature is included. Using the TreeExplainer module, we calculated precise SHAP values for the gradient boosting decision tree model. Visualization of the top features ranked by absolute mean SHAP values showed that specific acoustic features such as spectral entropy, energy change difference and speaking ratio have different contributions to predictions across the classes.

### 2.8. Statistical Analysis Environment

All statistical analysis and computations were conducted using SPSS version 26 and Python Version 3.6.2. Categorical variables were expressed as frequency (percentage). The continuous variables conforming to the normal distribution were expressed as the mean ± standard deviation and the other not conforming to normal distribution were presented as median (interquartile range). Differences with *p*-value < 0.05 were considered statistically significant. Bootstrap resampling was used solely to estimate the uncertainty of performance metrics and to derive confidence intervals, and it was not used for model selection, hyperparameter tuning, or statistical hypothesis testing.

## 3. Results

### 3.1. Patient Characteristics

**Figure 4:**
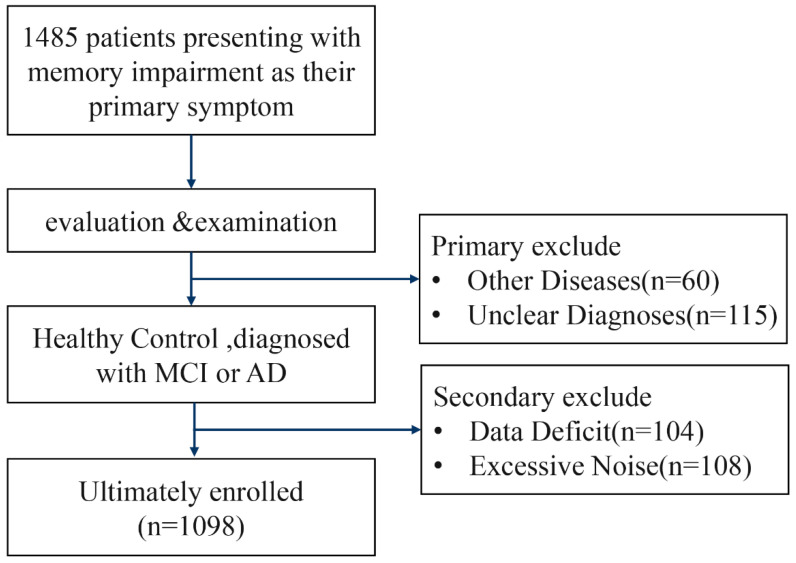
Flow diagram for patients’ enrollment.

Between 1 January 2023 and 31 December 2024, a total of 1485 patients were screened for inclusion in this study. Of these, 60 patients with other medical conditions and 115 patients with unknown diagnoses were excluded during the initial screening. An additional 104 patients were excluded due to inability or refusal to complete the full cognitive assessment or MRI examination. Furthermore, 108 patients were excluded because excessive noise in their voice recordings prevented the extraction of reliable acoustic features. Ultimately, 1098 patients met the inclusion criteria and were enrolled in this study. A detailed overview of the patient enrollment process is presented in Figure 4. Table 1 summarizes the clinical and sociodemographic characteristics of the sample used in this study.

### 3.2. Mel-Spectrogram and Spectral Analysis of Patient Speech

**Figure 5:**
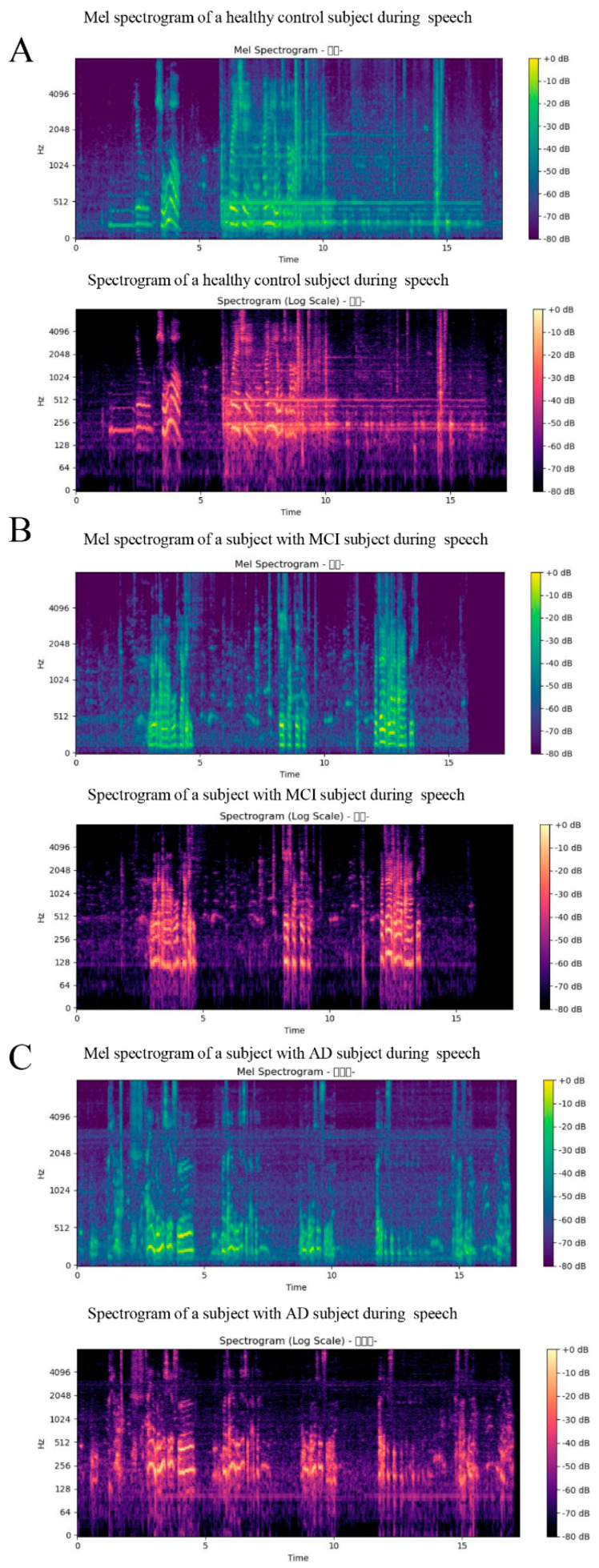
Mel spectrograms and spectral plots of speech samples from (**A**) healthy controls, (**B**) individuals with mild cognitive impairment (MCI), and (**C**) patients with Alzheimer’s disease (AD).

Figure 5A–C display the Mel-spectrograms and corresponding spectral graphs for participants in the healthy control group, those with mild cognitive impairment, and patients diagnosed with Alzheimer’s disease, respectively.

The Mel-spectrogram provides a time–frequency representation of the speech signal, highlighting energy distribution across Mel-scaled frequency bands. In Figure 5, comparative Mel-spectrograms are shown for healthy controls, MCI patients, and AD patients. Notably, AD patients exhibit sparse energy bands, irregular formant structures, and increased silence intervals, indicating reduced phonation consistency and impaired articulation. The corresponding spectral graphs illustrate the signal’s power spectral density. Compared to healthy controls, MCI and AD subjects show diminished spectral energy, particularly in the mid- to high-frequency ranges, reflecting weakened vocal strength and reduced clarity.

### 3.3. Performance Evaluation of Classification Models

**Figure 6:**
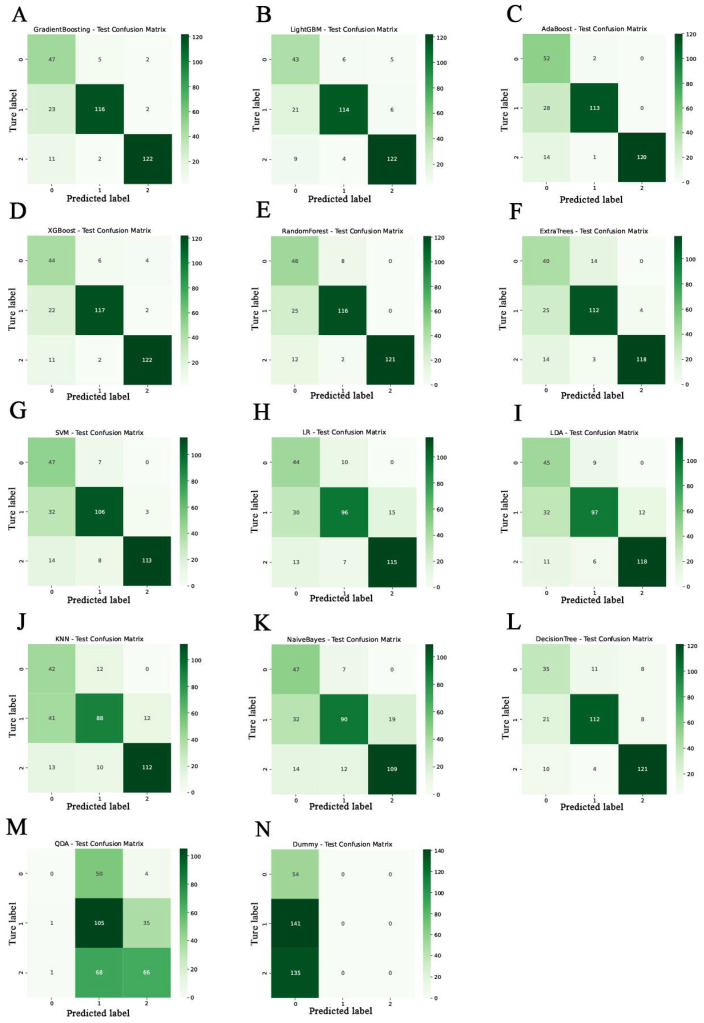
Confusion matrices of 14 classification models on the validation set. The models include: Gradient Boosting (**A**), Light Gradient Boosting Machine (LightGBM) (**B**), AdaBoost (**C**), eXtreme Gradient Boosting (XGBoost) (**D**), Random Forest (**E**), Extra Trees (**F**), Support Vector Machine (SVM) (**G**), Logistic Regression (LR) (**H**), Linear Discriminant Analysis (LDA) (**I**), k-Nearest Neighbors (KNN) (**J**), Naive Bayes (**K**), Decision Tree (**L**), Quadratic Discriminant Analysis (QDA) (**M**), and Dummy Classifier (**N**). Classification labels: 0—Healthy Control (HC), 1—Mild Cognitive Impairment (MCI), 2—Alzheimer’s Disease (AD).

To evaluate the effectiveness of various models in distinguishing between healthy controls, patients with mild cognitive impairment, and those with Alzheimer’s disease, we employed multiple performance metrics, including precision, recall, F1 score, and the area under the receiver operating characteristic curve. A total of 14 classification algorithms were assessed, ranging from conventional linear models to advanced ensemble learning techniques. Detailed summaries of the performance indicators for all model training and test sets can be found in Supplementary Material Tables S3 and S4, while Figure 6 presents the confusion matrices of each classifier for the test sets.

Among all tested models, Gradient Boosting (Figure 6A) and LightGBM (Figure 6B) exhibited the best overall performance. Gradient Boosting achieved the highest F1 score (0.837) and AUC (0.950), followed closely by LightGBM (F1 score: 0.816; AUC: 0.950). AdaBoost (Figure 6C) also demonstrated robust generalization capabilities, achieving an F1 score of 0.841 and an AUC of 0.926. Additionally, XGBoost (Figure 6D), Random Forest (Figure 6E) and ExtraTrees (Figure 6F) performed competitively, with AUC values of 0.945, 0.937 and 0.927, respectively.

In contrast, simpler models such as Support Vector Machine (Figure 6G), Logistic Regression (Figure 6H) and Linear Discriminant Analysis (Figure 6I) yielded moderate classification performance, with AUCs ranging from 0.87 to 0.90. K-Nearest Neighbors (Figure 6J), Naive Bayes (Figure 6K) and Decision Tree (Figure 6L) demonstrated relatively lower predictive accuracy and stability. Notably, Quadratic Discriminant Analysis (Figure 6M) and the Dummy (Figure 6N) classifier exhibited poor predictive performance, with AUCs of 0.579 and 0.500, respectively, indicating limited clinical utility in this context.

### 3.4. Feature Importance Analysis

To investigate the interpretability of model predictions across different cognitive states, we examined feature importance within the LightGBM and Gradient Boosting classifiers, focusing on their performance in distinguishing healthy controls (class 1), mild cognitive impairment (class 2), and Alzheimer’s disease (class 3).

**Figure 7:**
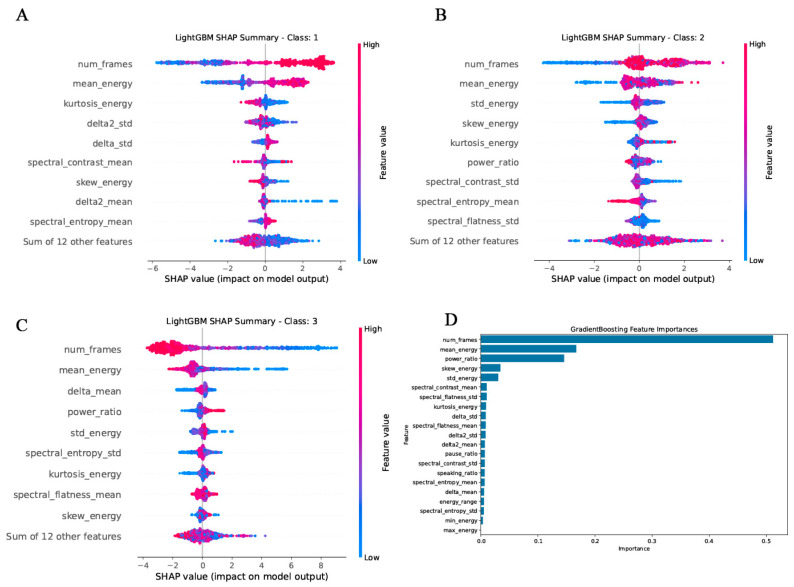
The importance of feature in optimized. (**A**) In the healthy controls group (class1). the SHAP values of each feature in the LightGBM model. (**B**) In the MCI group (class 2), the SHAP values of each feature in the LightGBM model. (**C**) In the AD group (class 3), the SHAP values of each feature in the LightGBM model. (**D**) Average SHAP value of each feature in the GradientBoosting.

Class-specific SHAP analyses derived from the LightGBM model revealed distinct feature patterns corresponding to HC, MCI and AD groups. For HC (Figure 7A), higher values of spectral entropy mean, delta2 mean, skew energy, and spectral contrast mean were the most predictive, indicating that cognitively healthy individuals tend to produce speech with higher spectral complexity, smoother energy distributions, and more structured spectral variation. Additionally, num frames, reflecting the total duration of spoken responses, contributed prominently, suggesting greater speech completeness and fluency in healthy subjects.

In the MCI group (Figure 7B), the model assigned greater importance to features indicative of disrupted vocal regularity and reduced spectral coherence. Variables such as spectral flatness std, spectral entropy mean, and spectral contrast std played key roles, reflecting increased noise components and weakened harmonic contrast. Furthermore, energy-based features including power ratio, kurtosis energy, std energy, and mean energy were found to be influential, potentially capturing the reduced vocal expressiveness and energetic variability associated with mild cognitive deterioration.

In the AD group (Figure 7C), the model places greater emphasis on acoustic indicators that reflect changes in temporal structure and energy characteristics during speech production. Features such as frame count, mean energy, and mean energy difference exhibited high importance in the SHAP analysis, indicating that speech duration and energy dynamics play a critical role in the model’s identification of this category, which may correspond to individuals with AD. In addition, energy-related features such as power ratio, energy standard deviation, and energy kurtosis showed notable influence, potentially reflecting reduced speech expressiveness and diminished energy control in patients with MCI and AD. Moreover, the model remains sensitive to spectral features, including the standard deviation of spectral entropy and the mean spectral flatness, suggesting a trend toward decreased spectral coherence and increased noise components during speech. Overall, the model tends to prioritize key parameters that reveal impairments in speech regulation and increased spectral complexity.

The complementary results from the Gradient Boosting model supported the findings from SHAP analysis (Figure 7D). The global feature importance ranking identified num frames and mean energy as the most discriminative features across the three-class classification task. These variables capture both the temporal length and vocal intensity of the utterances, reflecting macro-level speech production capacity. Other highly ranked features included power ratio, skew energy, and spectral contrast mean, alongside several dynamic descriptors such as delta std, delta2 mean, and delta2 std, all of which contribute to the model’s sensitivity to temporal fluctuations and phonatory control. The prominence of both static and dynamic acoustic parameters reinforces the notion that speech production is deeply intertwined with cognitive integrity, and that multidimensional vocal signals offer rich diagnostic information across the cognitive continuum.

## 4. Discussion

This study focuses on the intelligent identification of AD and MCI by developing a high-dimensional feature modeling and multi-algorithm classification framework based on a standardized MMSE speech task. This study utilized Mandarin MMSE speech recordings collected from the Sichuan-Chongqing region, encompassing three cohorts: HC, MCI, and AD patients. All speech samples underwent standardized preprocessing and manual annotation to ensure corpus quality and task consistency.

This work pioneers the integration of the pre-trained large language model CosyVoice2 into the AD speech recognition pipeline. Leveraging its self-supervised speaker embedding mechanism, high-dimensional frame-level speech representations (1024-dimensional vectors) were extracted to replace traditional low-dimensional handcrafted features such as MFCC, pitch, and speech rate. To mitigate the computational burden and reduce the risk of overfitting associated with directly modeling high-dimensional embeddings, we applied a statistical dimensionality reduction strategy. Specifically, various summary statistics such as mean, standard deviation, skewness, kurtosis, and the first and second derivatives were computed over the embedding sequences to generate interpretable feature vectors with reduced dimensionality. This approach preserves the semantic, cognitive, and speaker-related information encoded in deep speech embeddings while enhancing downstream model stability, controllability, and generalizability under limited sample conditions. We systematically evaluated the classification performance of 14 mainstream machine learning algorithms, including support vector machines, random forest, XGBoost, LightGBM, gradient boosting, k-nearest neighbors, and logistic regression. Five-fold cross-validation was used to compare their performance on the three-class classification task involving healthy controls, mild cognitive impairment, and Alzheimer’s disease. The Gradient Boosting and LightGBM models achieved high overall discrimination (AUC up to 0.95) despite F1 scores below 0.85, reflecting class imbalance and reduced sensitivity for the minority class. SHAP analyses of LightGBM highlighted features largely consistent with prior studies: HC exhibited higher spectral complexity and smoother energy distributions, MCI showed disrupted vocal regularity and reduced spectral coherence, and AD presented temporal and energy-related speech changes. Gradient Boosting analyses supported these patterns, with frame count and mean energy among the most discriminative features. Notably, less-studied features such as spectral contrast variability and pause ratio dynamics also contributed strongly, suggesting potential novel acoustic markers for cognitive decline. These findings reinforce known patterns while indicating new directions for speech-based cognitive assessment.

In recent years, speech has gained increasing attention as a non-invasive, low-cost, easily collectable, and reproducible digital biomarker for early screening of AD and MCI, drawing interest from both cognitive neuroscience and artificial intelligence communities [6,25,26,51]. Existing approaches primarily rely on low-dimensional handcrafted acoustic features, such as Mel-frequency cepstral coefficients, speech rate, fundamental frequency, and pause duration, in combination with classical classifiers such as support vector machines and random forest to model cognitive states [52,53,54]. For instance, Luz et al. developed SVM models based on rhythm and prosody features to identify AD individuals [55]; Martinc et al. incorporated MFCC and word embedding features within a multimodal fusion framework using RF classifiers [56]; Farrús et al. employed perturbation features such as jitter and shimmer to characterize speech differences between AD and HC groups [57]. To further characterize model behavior, we examined misclassification patterns using confusion matrices from the test set. Across multiple classifiers, MCI samples were the most frequently misclassified, often being assigned to either HC or AD. This pattern aligns with the clinical continuum of cognitive decline and indicates that boundary distinctions between adjacent categories remain challenging for automated models. Our results help explain why the multimodal speech representations improved model performance. The CosyVoice2 embeddings capture spectral, temporal, and prosodic cues that reflect cognitive processes, offering a more comprehensive description of speech than conventional handcrafted features. By integrating these complementary sources of information, the models can detect subtle abnormalities that may be missed by low-dimensional features alone, which likely accounts for the consistent performance gains across classifiers. Although these studies provide valuable insights for speech-based screening, they commonly suffer from several limitations: feature dimensionality remains low, limiting the capture of complex language behavior changes induced by cognitive impairment; models lack systematic semantic and speaker characteristic modeling, resulting in poor generalizability to non-standard language environments; moreover, heterogeneous evaluation metrics and task settings hinder comparability and reproducibility across studies.

To address these challenges, the present study introduces key innovations in feature modeling, algorithm integration, and task standardization. CosyVoice2 is a pre-trained large language model developed by Alibaba for text-to-speech and speech generation tasks [29]. It demonstrates strong capabilities in speaker disentanglement and semantic modeling. The model shows high adaptability in the context of the Sichuan dialect, effectively identifying and capturing subtle and complex variations in language behavior. Compared to traditional handcrafted acoustic metrics, its high-dimensional embeddings provide more abstract and cognition-sensitive representations without requiring explicit feature engineering. We simplified the extracted high-dimensional feature sequences into interpretable and structurally concise features that are compatible with various machine learning models, including SVM, gradient boosting, Decision Trees and LightGBM. This “semantic embedding plus statistical modeling” fusion strategy enhances model generalization across languages and populations, demonstrating improved stability under small-sample conditions. Lastly, leveraging a standardized MMSE speech task and a systematic comparative evaluation framework, this study establishes a replicable and scalable technical pipeline with benchmark metrics for future speech-assisted cognitive impairment screening.

Despite promising advances in both methodological approach and classification performance, several limitations remain. Our study has several practical implications for clinical application. The proposed speech-based assessment framework could be integrated into routine cognitive screening workflows, allowing non-specialist staff to collect and analyze speech samples with minimal disruption to clinical operations. However, deploying the framework in real-world clinical settings may face challenges, including limited computational resources for processing high-dimensional embeddings, variability in recording equipment, and the influence of environmental noise. Potential biases may also arise from differences in participant demographics such as age, education, or language dialect, as well as heterogeneous recording conditions across sites. Implementing standardized recording protocols, robust model calibration, and external validation across diverse populations will be essential to ensure reliable and generalizable performance in practical clinical environments. The trained models are geographically and linguistically constrained by the Sichuan-Chongqing data, necessitating further validation across multilingual and multi-regional cohorts. Additionally, clinical cognitive labels based on neuropsychological scale scores may be subject to subjective assessment bias, warranting incorporation of objective biomarkers such as neuroimaging or biofluid indicators in future annotation schemes. Furthermore, this study has yet to integrate multimodal physiological data, leaving room for improvement in modeling the complexity of cognitive function.

Future work will include a systematic comparison between CosyVoice2 embeddings and conventional handcrafted acoustic features, such as MFCCs, pitch, and formant-related measures, using the same classification framework. This analysis will help clarify the relative contributions of learned speech representations and downstream classifiers. We also plan to explore feature fusion strategies to assess potential complementarity between learned and traditional acoustic features. We will focus on enhancing the reliability and applicability of our approach. This includes robustness testing under varied recording conditions, evaluating generalizability across independent multi-center datasets, and validating the framework in real-world clinical settings to assess feasibility, workflow integration, and practical utility. These steps aim to ensure consistent and reliable performance in diverse real-world conditions. Furthermore, prospective validation in multicenter clinical settings is essential to translate this research-driven approach into practical clinical decision support systems.

## 5. Conclusions

In conclusion, this study proposes a robust and scalable framework for Alzheimer’s disease screening that addresses key limitations of existing methods, including poor dialect adaptability and low feature expressiveness. By combining high-dimensional acoustic embeddings from pre-trained language models with structured language tasks, the approach effectively captures cognitive speech impairments across diverse dialects. The results support its potential as a non-invasive digital biomarker for early detection and broad clinical application.

## Figures and Tables

**Table 1:** Participants’ demographic characteristics.

Variable	Total N = 1098	HC N = 179	MCI N = 470	AD N = 449	*p*-Value
Age	73.56 (11.6)	73.00 (13)	74.00 (11)	73.00 (10)	0.573 ^a^
Female, N (%)	738 (67.213)	119 (66.48)	307 (65.31)	312 (69.49)	0.394 ^b^
Education, (y)	8.62 (6.222)	9.000 (6.0)	9.000 (6.3)	9.000 (6.0)	0.632 ^a^
MMSE	26.05 (12.52)	28.00 (2)	22.00 (12)	19.00 (15)	<0.001 ^a^*
ADAS-Cog	13.0574 (17.4433)	8.300 (5.9)	15.315 (16.08)	18.7100 (26.03)	<0.001 ^a^*

Data are presented as median (interquartile range); ^a^, Kruskal–Wallis test; ^b^, the chi-square test; *, *p* < 0.05. Abbreviation: HC healthy controls, MCI mild cognitive impairment, AD Alzheimer’s disease, MMSE Mini-mental State Examination, ADAS-Cog Alzheimer’s Disease Assessment Scale-Cognitive section.

## Data Availability

The datasets generated and/or analyzed during the current study are not publicly available due to the presence of sensitive personal data, but are available from the corresponding authors upon reasonable request.

## Supplementary Materials

The following supporting information can be downloaded at: https://www.mdpi.com/article/10.3390/bioengineering13010073/s1. Table S1. Overview and explanation of extracted speech features. Table S2. Demographic characteristics of the training set and the test set. Table S3. Performance evaluation table of Machine learning model in train set. Table S4. Performance evaluation table of Machine learning model in test set. Table S5. Mapping of clinical constructs to extracted speech features. References [26,58,59,60,61] are cited in supplementary materials.
