# Supplementary material for: A Machine Learning Framework for Cognitive Impairment Screening from Speech with Multimodal Large Models
Source: Bioengineering (Basel). 2026 Jan 8;13(1):73. doi: 10.3390/bioengineering13010073 (PMC12837762; doi:10.3390/bioengineering13010073)
Supplement: Supplementary file 1 [file bioengineering-13-00073-s001.zip › bioengineering-4013735-supplementary.pdf]

Supplementary Materials

Table S1. Overview and explanation of extracted speech features.

Speech features	Explanation
Num frames	Total number of audio frames analyzed
Mean energy	Average energy of the signal, reflecting vocal intensity
Std energy	Standard deviation of energy across frames, indicating variability
Max energy	Maximum frame-level energy observed
Min energy	Minimum frame-level energy observed
Energy range	Difference between max and min energy, representing dynamic vocal range
Skew energy	Skewness of the energy distribution, indicating asymmetry
Kurtosis energy	Kurtosis of the energy distribution, reflecting peakedness
Delta mean	Mean of the first-order derivative, measuring temporal change in energy
Delta std	Standard deviation of first-order derivative features
delta2 mean	Mean of the second-order derivative, reflecting acceleration in energy change
delta2 std	Standard deviation of second-order derivative

Power sum	Total power over the signal duration
Power ratio	Ratio of power in voiced vs. unvoiced segments
Pause ratio	Proportion of time spent in silence
Speaking ratio	Proportion of time spent speaking
Spectral entropy mean	Mean of spectral entropy, measuring signal complexity
Spectral entropy std	Standard deviation of spectral entropy
Spectral flatness mean	Mean of spectral flatness, indicating tonality vs. noisiness
Spectral flatness std	Standard deviation of spectral flatness
Spectral contrast mean	Average spectral contrast across frequency bands
Spectral contrast std	Variability in spectral contrast

Table S2. Demographic characteristics of the training set and the test set.

Variable	Train N=769	Test N=329	*p*-Value
Age	73.00（11）	74.00（12）	0.51 [a*]
Female, N (%)	510（66.32）	228(69.30)	0.335[b]
Education, (y)	9.000（6.0）	9.000（7.0）	0.087 [a*]
MMSE	25.00（13）	27.00（13）	0.356 [a*]
ADAS-Cog	13.00（19）	13.00(14.97)	0.864 [a*]

Data are presented as median (interquartile range); [a], Kruskal–Wallis test; [b], the chi-squared test; *, $p < 0.05$. Abbreviation: NC normal cognition, MCI mild cognitive impairment, ADD dementia due to Alzheimer's disease, MMSE Mini-mental State Examination, ADAS-Cog Alzheimer's Disease Assessment Scale-Cognitive section.

Table S3. Performance evaluation table of Machine learning model in train set.

Model	Precision（95%CI)	Recall（95%CI)	F1 Score（95%CI)	AUC（95%CI)
LR	0.780（0.758,0.802)	0.816（0.796,0.836)	0.789（0.768,0.808)	0.926（0.912,0.940)
RandomForest	0.842（0.826,0.858)	0.864（0.849,0.879)	0.849（0.833,0.865)	0.949（0.936,0.962)
ExtraTrees	0.809（0.792,0.826)	0.813（0.796,0.830)	0.809（0.786,0.828)	0.945（0.931,0.959)
GradientBoosting	0.824（0.808,0.840)	0.845（0.831,0.859)	0.831（0.812,0.848)	0.949（0.936,0.962)
AdaBoost	0.854（0.839,0.869)	0.890（0.876,0.904)	0.862（0.845,0.878)	0.941（0.929,0.953)
KNN	0.722（0.700,0.744)	0.753（0.732,0.774)	0.722（0.703,0.744)	0.885（0.868,0.902)
DecisionTree	0.801（0.785,0.817)	0.806（0.789,0.823)	0.803（0.787,0.818)	0.873（0.855,0.891)
SVM	0.793（0.777,0.809)	0.828（0.813,0.843)	0.800（0.781,0.818)	0.930（0.915,0.945)
NaiveBayes	0.719（0.697,0.741)	0.753（0.732,0.774)	0.728（0.708,0.747)	0.879（0.862,0.896)
LDA	0.789（0.773,0.805)	0.826（0.811,0.841)	0.797（0.779,0.815)	0.926（0.912,0.940)
QDA	0.524（0.494,0.554)	0.508（0.479,0.537)	0.450（0.430,0.470)	0.612（0.583,0.641)
XGBoost	0.825（0.809,0.841)	0.846（0.831,0.861)	0.832（0.813,0.849)	0.948（0.935,0.961)

LightGBM	0.841（0.826,0.856）	0.86（0.847,0.877）	0.849（0.832,0.865）	0.956（0.944,0.968）
Dummy	0.054（0.035,0.073）	0.333（0.303,0.363）	0.093（0.035,0.160）	0.5（0.459,0.541）

Table S4. Performance evaluation table of Machine learning model in test set.

Model	Precision	Recall	F1 Score	AUC
GradientBoosting	0.831（0.799, 0.863）	0.866（0.839, 0.893）	0.837（0.799, 0.878）	0.950（0.932, 0.968）
LightGBM	0.809（0.774, 0.844）	0.836（0.806, 0.866）	0.816（0.774, 0.855）	0.950（0.932, 0.968）
AdaBoost	0.842（0.811, 0.873）	0.884（0.858, 0.910）	0.841（0.811, 0.891）	0.926（0.904, 0.948）
XGBoost	0.820（0.786, 0.854）	0.849（0.820, 0.878）	0.826（0.786, 0.865）	0.945（0.926, 0.964）
RandomForest	0.825（0.791, 0.859）	0.857（0.829, 0.885）	0.829（0.791, 0.872）	0.937（0.916, 0.958）
ExtraTrees	0.781（0.744, 0.818）	0.803（0.772, 0.834）	0.783（0.744, 0.820）	0.927（0.905, 0.949）
SVM	0.785（0.748, 0.822）	0.820（0.790, 0.850）	0.783（0.748, 0.825）	0.917（0.894, 0.940）
LR	0.747（0.707, 0.787）	0.783（0.751, 0.815）	0.749（0.707, 0.790）	0.903（0.878, 0.928）
LDA	0.762（0.723, 0.801）	0.798（0.767, 0.829）	0.764（0.723, 0.806）	0.901（0.876, 0.926）
KNN	0.714（0.673, 0.755）	0.744（0.710, 0.778）	0.709（0.673, 0.755）	0.880（0.852, 0.908）
NaiveBayes	0.728（0.688, 0.768）	0.772（0.739, 0.805）	0.729（0.688, 0.772）	0.871（0.842, 0.900）
DecisionTree	0.765（0.726, 0.804）	0.780（0.748, 0.812）	0.770（0.726, 0.804）	0.844（0.812, 0.876）

QDA	0.366（0.322, 0.410）	0.411（0.366, 0.456）	0.376（0.322, 0.456）	0.580（0.533, 0.627）
Dummy	0.055（0.033, 0.077）	0.333（0.291, 0.375）	0.094（0.033, 0.160）	0.5（0.452, 0.548）

Table S5. Mapping of clinical constructs to extracted speech features.

Clinical Construct	Description	Speech Features Extracted
Verbal Fluency [1]	Ability to produce words fluently	Num frames, Speaking ratio, Pause ratio, Delta mean, Delta std
Articulation Precision [2]	Articulation Precision	Mean energy, Std energy, Max energy, Min energy, Energy range, Skew energy, Kurtosis energy
Prosody / Intonation [3]	Pitch, stress, and rhythm variations	Delta2 mean, Delta2 std, Power sum, Power ratio, Spectral contrast mean, Spectral contrast std
Memory Recall [4]	Ability to recall words or sentences	Num frames, Speaking ratio, Pause ratio, Spectral entropy mean, Spectral entropy std
Attention/Concentration [4]	Sustained focus reflected in speech patterns	Power ratio, Pause ratio, Spectral flatness mean, Spectral flatness std

Language Comprehension [5]	Understanding and responding to verbal prompts	Spectral entropy mean, Spectral entropy std, Spectral flatness mean, Spectral flatness std

[1] H.-L. Wang, R. Tang, R.-J. Ren, E.B. Dammer, Q.-H. Guo, G.-P. Peng, H.-L. Cui, Y.-M. Zhang, J.-T. Wang, X.-Y. Xie, Q. Huang, J.-P. Li, F.-H. Yan, S.-D. Chen, N.-Y. He, G. Wang, Speech silence character as a diagnostic biomarker of early cognitive decline and its functional mechanism: a multicenter cross-sectional cohort study, BMC Med 20 (2022) 380. https://doi.org/10.1186/s12916-022-02584-x.

[2] S. Saeedi, S. Hetjens, M.O.W. Grimm, B. Barsties V. Latoszek, Acoustic Speech Analysis in Alzheimer's Disease: A Systematic Review and Meta-Analysis, The Journal of Prevention of Alzheimer's Disease 11 (2024) 1789–1797. https://doi.org/10.14283/jpad.2024.132.

[3] C. Themistocleous, M. Eckerström, D. Kokkinakis, Voice quality and speech fluency distinguish individuals with Mild Cognitive Impairment from Healthy Controls, PLoS ONE 15 (2020) e0236009. https://doi.org/10.1371/journal.pone.0236009.

[4] V.D. Badal, J.M. Reinen, E.W. Twamley, E.E. Lee, R.P. Fellows, E. Bilal, C.A. Depp, Investigating Acoustic and Psycholinguistic Predictors of Cognitive Impairment in Older Adults: Modeling Study, JMIR Aging 7 (2024) e54655. https://doi.org/10.2196/54655.

[5] S. Cho, N. Nevler, S. Shellikeri, N. Parjane, D.J. Irwin, N. Ryant, S. Ash, C. Cieri, M. Liberman, M. Grossman, Lexical and Acoustic Characteristics of Young and Older Healthy Adults, J Speech Lang Hear Res 64 (2021) 302–314. https://doi.org/10.1044/2020_JSLHR-19-00384.
